# Coevolution of non-fertile sperm and female receptivity in a butterfly

**DOI:** 10.1098/rsbl.2009.0452

**Published:** 2009-07-29

**Authors:** Nina Wedell, Christer Wiklund, Jonas Bergström

**Affiliations:** 1 School of Biosciences, University of Exeter, Cornwall Campus, Penryn TR10 9EZ, UK; 2 Department of Zoology, Stockholm University, Stockholm S-106 91, Sweden

**Keywords:** sexual conflict, sperm competition, coevolution

## Abstract

Sexual conflict can promote rapid evolution of male and female reproductive traits. Males of many polyandrous butterflies transfer nutrients at mating that enhances female fecundity, but generates sexual conflict over female remating due to sperm competition. Butterflies produce both normal fertilizing sperm and large numbers of non-fertile sperm. In the green-veined white butterfly, *Pieris napi*, non-fertile sperm fill the females' sperm storage organ, switching off receptivity and thereby reducing female remating. There is genetic variation in the number of non-fertile sperm stored, which directly relates to the female's refractory period. There is also genetic variation in males' sperm production. Here, we show that females' refractory period and males' sperm production are genetically correlated using quantitative genetic and selection experiments. Thus selection on male manipulation may increase the frequency of susceptible females to such manipulations as a correlated response and *vice versa*.

## 1. Introduction

Sexual conflict occurs in sexually reproducing organisms and may promote rapid antagonistic coevolution of male and female reproductive traits. This is particularly true for traits involved in conflict over female mating in promiscuous species, where males manipulate female receptivity and females resist manipulations. There is evidence of rapid evolution of traits involved in sexual conflict (Arnqvist & Rowe 2005). However, sexual conflict will not invariably generate rapid evolution (Lessells 2006; Tregenza *et al*. 2006). The outcome critically depends on which sex controls mating decisions (Hosken *et al*. 2009), and the extent to which sexual conflict generates selection (Parker 2006).

One important aspect affecting the rate of sexual coevolution is the genetic correlation between reproductive traits (Lande 1981). Under sexual conflict, a negative genetic correlation between fitness-related traits is predicted between the sexes. Experimental evolution studies have demonstrated genetic associations between male manipulative traits and female response traits, implying genetic correlations between these traits (e.g. Holland & Rice 1999; Martin & Hosken 2003). Commonly, monogamy is enforced in a promiscuous species resulting in benign males and less-resistant females, indicative of negative genetic correlations between the traits involved. However, there is a dearth of studies specifically documenting the underlying genetic architecture of the traits in question.

In the polyandrous green-veined white butterfly *Pieris napi*, there is sexual conflict over female mating rate, which is exacerbated by male nutrient provisioning. Polyandrous females have higher reproductive output, whereas males attempt to impose monogamy to avoid sperm competition (Cook & Wedell 1999; Wedell *et al*. 2002). Males (like all butterflies) transfer two types of sperm: fertile (eupyrene) and non-fertile (apyrene) sperm that fills the females' sperm storage organ and switches off female receptivity (Cook & Wedell 1999). There is genetic variation in the females' refractory period, which is directly related to the number of non-fertile sperm stored (Wedell 2001). There is also genetic variation in males' sperm production (Wedell 2001). Sexual conflict over female receptivity in *P. napi* thus involves production and storage of non-fertile sperm, and may be responsible for the ejaculate consisting predominantly of non-fertile sperm. Here we examine the genetic architecture of female refractory period and non-fertile sperm transfer in *P. napi* to determine the potential for these traits to coevolve.

## 2. Material and methods

### (a) Insect husbandry

Adult females were captured in Stockholm, Sweden. Thirty offspring from each female were reared in sub-groups of five on *Alliaria petiolata* leaves at 24°C on a 22 L : 2 D cycle. On the morning after eclosion, individuals were weighed and given a colour mark to assign them to their family of origin. In total, offspring from 31 wild-caught females were reared. This procedure was repeated with 28 wild-caught females at a later date. The offspring were either assigned to a half-sibling/full-sibling breeding design to calculate heritabilities (see below), or used to examine correlations between the sexes across full-sibling families (*n* = 25 families).

### (b) Female refractory period

At 1 day of age, female offspring from the half-sibling (see below), or full-sibling families were haphazardly mated to a 1-day-old unrelated virgin male. Mating takes an average of 90 min. Following mating, females were provided with virgin (unrelated) males, *A. petiolata* for oviposition, and allowed to remate up to 10 days after their first mating. Females will rarely remate after this time (Wedell *et al*. 2002). The refractory period (the number of days between first and second mating) was noted.

### (c) Sperm counts

Male *P. napi*'s transfer two types of sperm in the spermatophore at mating; fertile, eupyrene, sperm and a large number of non-fertile, apyrene sperm. Non-fertile sperm are morphologically distinct from fertile sperm, and constitute more than 90 per cent of total sperm number (Cook & Wedell 1996). At 1 day of age, virgin males were haphazardly assigned to unrelated virgin females and allowed to mate. Females were frozen immediately after the end of copulation and the number of fertile and non-fertile sperm present in the males' first spermatophore were measured following a standard protocol (Cook & Wedell 1996).

### (d) Family mean correlations

The relationship between the number of apyrene and eupyrene sperm present in the males' first spermatophore and the female refractory period was examined across full-siblings from 25 families (mean of three sons and three daughters/family) using Spearman rank correlations corrected for ties.

### (e) Genetic correlations

Heritabilities of the number of fertile and non-fertile sperm (*n* = 17 sires) and the female refractory period (*n* = 12 sires) were estimated from the full-sibling/half-sibling design (three dams/sire with two to three offspring scored). Analyses of genetic variations were conducted on sire and dam variance components estimated with restricted maximum likelihood (SPSS v. 16.0), and *G*-tests used to test the significance of the sire estimates. Genetic correlations between the refractory period and sperm numbers were calculated from covariances estimated from a multivariate-nested ANOVA (*n* = 17 (sperm numbers) or 12 (refractory period) sires each mated to three dams and two to three offspring scored per dam).

### (f) Selection lines

Two lines were established from 51 wild-caught females as above. One-day-old virgin females were allowed to mate and lay eggs for 2 days, before being allowed to remate and the refractory period noted. The eggs laid before remating of the first eight females to remate founded the high mating rate line (‘polyandry’), and the eggs from eight females that did not remate during this time founded the low remating rate line (‘monogamy’). This procedure was repeated at each generation (scoring 30–40 females per selection line), but with butterflies mating within their own selection regime (see Bergström 2004). After eight generations of selection, the number of sperm was determined as above. The impact of female selection history on males' sperm transfer was analysed using generalized linear models (Crawley 2005), specifying a Poisson error distribution (data corrected for over-dispersion).

## 3. Results

### (a) Female refractory period

Female refractory period was heritable in *P. napi* (*h*^2^ = 0.772 ± 0.350, *G* = 4.244, *p* = 0.039; table 1). There was no effect of female weight (*p* > 0.2) or weight of the first male (*p* > 0.4, *n* = 101) on the refractory period.

**Table 1.** Cross-sire family means from multivariate-nested ANOVA (*r*) and genetic correlations (in bold, mean ± s.e., upper panel). *p < 0.05; **p < 0.001; ***p < 0.0005; n.s. = not significant.

	male weight	refractory period	eupyrene	apyrene
male weight	–	**n.s.**	**n.s.**	**n.s.**
refractory period	*r* = 0.125, *p* > 0.3	–	0.501 ± 0.376*	0.480 ± 0.373*
eupyrene sperm	*r* = 0.285, *p* > 0.1	*r* = 0.590*	–	0.922 ± 0.137*
apyrene sperm	*r* = 0.066, *p* > 0.9	*r* = 0.696**	*r* = 0.848***	–

### (b) Male sperm production

The number of sperm transferred was heritable. This was true for both fertile (*h*^2^ = 0.840 ± 0.411, *G* = 4.353, *p* = 0.003) and non-fertile sperm (*h*^2^ = 0.424 ± 0.281, *G* = 2.074, *p* = 0.042). There was no relationship between the number of either fertile or non-fertile sperm and male size (table 1).

### (c) Genetic correlations

**Figure 1.**
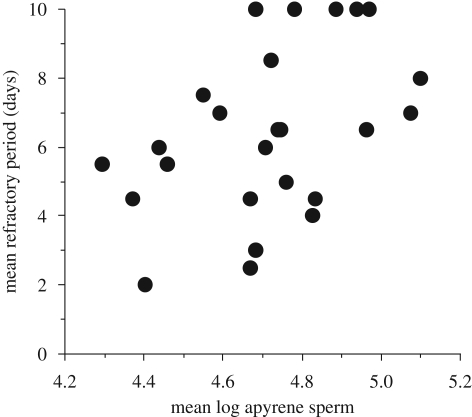
The relationship across full-sibling families between the mean duration of the female refractory period and the mean number of non-fertile apyrene sperm (log 10(mean)) transferred by males (*r*_s_ = 0.49, *z* = 2.401, *p* = 0.017, *n* = 25 families).

The female refractory period is directly related to the number of non-fertile sperm stored (Cook & Wedell 1999; Wedell 2001). Full-sibling analysis revealed a positive relationship between the mean refractory period of females and the average number of non-fertile sperm (figure 1), but no significant relationship between the mean refractory period of females and the number of fertile sperm (*r*_s_ = 0.30, *z* = 1.474, *p* > 0.1). This relationship was also confirmed by a genetic correlation between number of non-fertile sperm and the female refractory period in the half-sibling analysis (*r*_G_ = 0.480 ± 0.373, *p* < 0.05). There was also a genetic correlation between the number of fertile sperm and the refractory period (*r*_G_ = 0.501 ± 0.376, *p* < 0.05).

### (d) Selection lines

**Figure 2.**
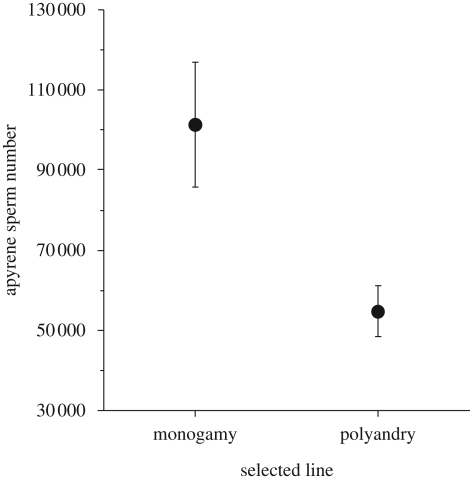
The number of non-fertile apyrene sperm transferred by males in relation to the selection regime experienced. Males from the polyandrous line where females had been selected for short refractory periods transferred fewer numbers of non-fertile sperm (*F*_1,51_ = 13.539, *p* = 0.0006, *n* = 36 ‘polyandry’; *n* = 17 ‘monogamy’) than males from the monogamy line where females were selected for long refractory periods. Means ± s.e.

The selection lines also provided evidence of a genetic correlation between male sperm transfer and female refractory period. Males from the line where females were selected for slow remating rates transferred significantly more fertile (*F*_1,51_ = 19.437, *p* < 0.0001) and non-fertile sperm (figure 2). This indicates that selection on female refractory period can promote changes in males' sperm production, although genetic drift cannot be ruled out owing to lack of line replication.

## 4. Discussion

Non-fertile sperm transfer and female refractory period is positively genetically correlated in *P. napi* as revealed by three separate studies: full-sibling family mean correlations, half-sibling quantitative genetic analyses, and the selection experiment reported here. Female refractory period is also genetically correlated with fertile sperm transfer, but was not correlated across full-sibling families. There is therefore scope for selection acting on females' receptivity and non-fertile sperm transfer, and evolutionary responses to selection in these traits. This genetic correlation is probably owing to linkage disequilibrium caused by males producing many non-fertile sperm, increasing the refractory period of females that store many non-fertile sperm and *vice versa*. Sexual conflict over female remating rate in *P. napi* thus involves non-fertile sperm, as they switch off female receptivity (Cook & Wedell 1999), despite direct benefits from polyandry to females (Wedell *et al*. 2002), and may explain why non-fertile sperm make up 90 per cent of total sperm number.

Most analyses of sexual conflict over female mating explore situations when mating is costly to females. By contrast, *P. napi* females benefit from polyandry owing to male nutrient donations, although monogamous females live longer than genetically polyandrous females prevented from remating (Wedell *et al*. 2002). The mating conflict involves male manipulation (non-fertile sperm transfer) and female resistance (non-fertile sperm storage). It is unknown what the fitness costs are to males of producing many or few sperm.

Models exploring the potential for sexual conflict to generate antagonistic coevolution stress the importance of the shape of the females' response to male manipulation. Exaggeration of male traits involved in overcoming female resistance is sensitive to the shape of the response in female resistance. If females increase the threshold amount of male stimulation (i.e. non-fertile sperm) required to switch off mating, this can generate cycles of coevolution. By contrast, if females evolve to become insensitive to males' manipulation, they no longer exert selection on males and hence there is no evolution (Rowe *et al*. 2005). The outcome depends on the genetic variance in female resistance traits and the strength of natural selection acting on the trait(s) (Rowe *et al*. 2005). While it is clear that there is substantial genetic variation in the female refractory period in *P. napi*, it is not known to what extent storage of non-fertile sperm is subject to natural selection, but it is possible that non-fertile sperm may affect female overall fertility. The relationship between transfer and storage of non-fertile sperm is also complex. The numbers stored are substantially more variable than the number of non-fertile sperm inseminated (Wedell 2001).

The finding that female refractory period and sperm transfer are genetically correlated in *P. napi* is consistent with the previous findings showing that selection on female reproductive traits can directly affect male traits and *vice versa* (Martin & Hosken 2003). Coevolution between male and female reproductive traits (i.e. sperm production and storage) is unlikely to be affected by indirect genetic effects (i.e. females siring manipulative sons), as direct benefits are generally greater in magnitude (Cameron *et al*. 2003). The benefit to female *P. napi* of multiple mating in terms of increased fecundity vastly outweigh any potential benefit of siring manipulative sons that are better at reducing female receptivity. It is also unlikely owing to sperm production being a condition-dependent trait, as larval diet only affects males' nutrient donation but not sperm numbers (Cook & Wedell 1996), and diet does not influence females' likelihood of remating (Bergström & Wiklund 2002). Thus, sexual conflict is a likely candidate for the observed genetic correlation between female refractory period and male non-fertile sperm production in this butterfly.
